# Testosterone regulates the autophagic clearance of androgen binding protein in rat Sertoli cells

**DOI:** 10.1038/srep08894

**Published:** 2015-03-09

**Authors:** Yi Ma, Hao-Zheng Yang, Long-Mei Xu, Yi-Ran Huang, Hui-Li Dai, Xiao-Nan Kang

**Affiliations:** 1 Department of Biobank, Renji Hospital, School of Medicine, Shanghai JiaoTong University, Building 1, 1630 DongFang Road, Shanghai. 200127, China; 2 Department of Urology, Renji Hospital, School of Medicine, Shanghai JiaoTong University, Building 7, 1630 DongFang Road, Shanghai. 200127, China; 3 Department of Central Laboratory, Renji Hospital, School of Medicine, Shanghai JiaoTong University, Building 5, 1630 DongFang Road, Shanghai. 200127, China

## Abstract

Dysregulation of androgen-binding protein (ABP) is associated with a number of endocrine and andrology diseases. However, the ABP metabolism in Sertoli cells is largely unknown. We report that autophagy degrades ABP in rat Sertoli cells, and the autophagic clearance of ABP is regulated by testosterone, which prolongs the ABP biological half-life by inhibiting autophagy. Further studies identified that the autophagic clearance of ABP might be selectively regulated by testosterone, independent of stress (hypoxia)-induced autophagic degradation. These data demonstrate that testosterone up-regulates ABP expression at least partially by suppressing the autophagic degradation. We report a novel finding with respect to the mechanisms by which ABP is cleared, and by which the process is regulated in Sertoli cells.

Sex hormone-binding globulin (SHBG) is a glycoprotein that is mostly produced by the liver, testes, brain, and placenta. Testes-produced SHBG is also called androgen-binding protein (ABP). ABP specifically binds to testosterone or dihydrotestosterone, making them less lipophilic and more concentrated in the seminiferous tubules. High levels of ABP are essential for maintaining the microenvironment as well as enable spermatogenesis in the seminiferous tubules and sperm maturation in the epididymis. ABP can promote germ cell differentiation, and it has been reported that ABP up-regulates the expression of transition protein 1 (TP1), which is involved in nuclear chromatin condensation in rodent spermatids^1^. ABP can also directly up-regulate the expression of aromatase in germ cells and oestrogen receptor beta^2^. In humans, an alternative ABP transcript accumulates in germ cells in a highly regulated manner throughout the spermatogenic cycle^3^, and its expression is positively correlated with sperm motility^4^. It has been reported that human ABP transcription is controlled by several germ cell-enriched transcription factors, including SPZ1 and CREB/CREM^5^. When ABP migrates together with immature sperm to the caput epididymis, it promotes the access of testosterone to the immature sperm and promotes sperm maturation^6^. ABP also aids in the transport of testosterone, dihydrotestosterone and oestrogens throughout the body, which continue to play a role in the target organs. The affinity of ABP is four to five orders of magnitude greater than that of albumin; therefore, it enhances the solubility of those lipophilic molecules and prolongs their biological half-life.

The synthesis and secretion of ABP is regulated by follicle-stimulating hormone (FSH), testosterone, oestradiol, and other factors^7^,^8^,^9^. Aberrant control of ABP production has been found in several endocrine and andrology disorders^10^,^11^. Varicocele-induced dysregulation of ABP may be a parameter of impaired reproduction function^10^, and significantly lower ABP secretion in non-obstructed azoospermic (NOA) patients compared with obstructed azoospermic (OA) patients may indicate a defect in ABP synthesis in NOA patients^11^. However, it is still not clear how the process of ABP metabolism works in Sertoli cells or how the process is regulated.

Autophagy is active in Sertoli cells^12^,^13^. Autophagy is a lysosomal degradation pathway that eliminates defective organelles and proteins from the cell. Classic macroautophagy is initiated from an isolated membrane (phagophore), which is followed by the formation of a double-membrane autophagosome that then delivers the engulfed proteins and fuses with lysosomes for degradation. MTOR signalling and ATG family members strictly regulate autophagy. In Sertoli cells, autophagy can serve as a cell survival pathway or cooperate with apoptosis to promote cell death^14^,^15^,^16^, in addition to degrading ingested cell-derived substrates^13^. However, the function of autophagy in Sertoli cells is largely unknown.

In this study, we have revealed that autophagy clears ABP in rat Sertoli cells, and this process appears to be selectively regulated by testosterone and independent of stress (hypoxia)-induced autophagic degradation.

## Results

### Autophagy regulates the ABP expression level in rat Sertoli cells

**Figure 1:**
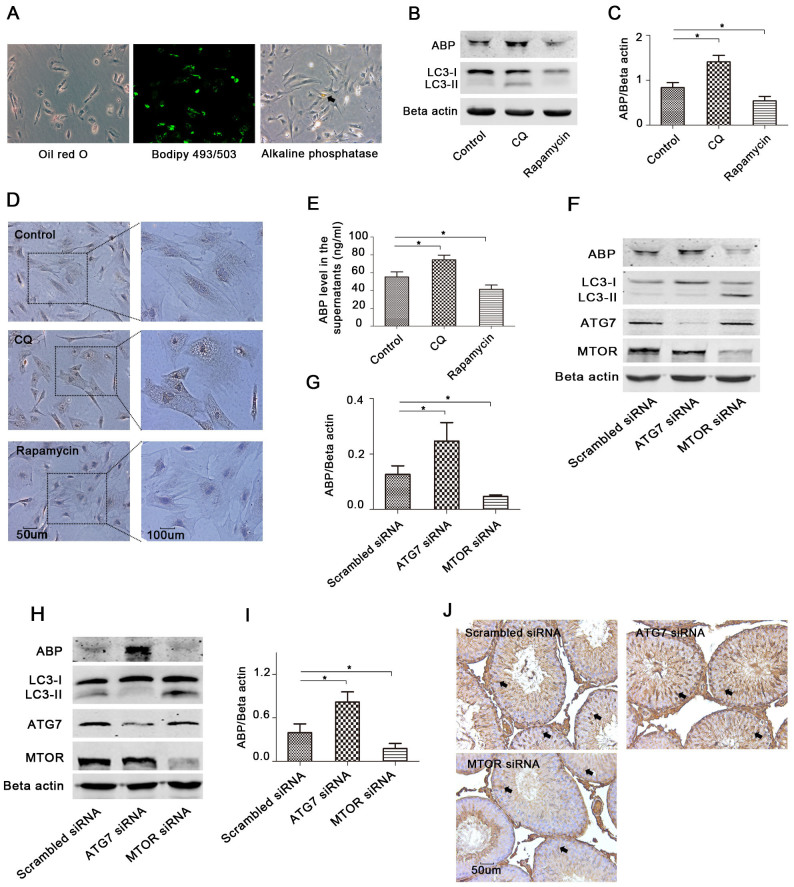
Autophagy regulates ABP expression in the primary Sertoli cells and rat testes. (A), Cells isolated from 20-day-old rat testis were stained with oil red O, bodipy 493/503 or alkaline phosphatase detection kit, and pictures were taken with a fluorescence microscope. Black arrow: germ cell. (B–E), Rat primary Sertoli cells were treated with CQ (50 uM) or rapamycin (10 nM) for 24 h, and ABP expression in the cells was assessed with Western blot (B) and immunocytochemistry (D). ABP in the supernatants was determined by ELISA (E) (n = 3, *p < 0.05). Densitometric analysis of the bands in ABP is shown in (C) (mean ± SD of independent experiments, n = 3, *p < 0.05). LC3B was assessed by Western blot (B). (F–G), Cells were treated with scrambled siRNA, ATG7 siRNA, or MTOR siRNA for 48 h, and ABP, LC3B, ATG7, MTOR and Beta actin were determined by Western blots (F), and densitometric analysis of ABP immunoblots is shown (G) (n = 3, *p < 0.05). (H–J), The rat testis was directly injected with 50 ul mixed liquid of in vivo-jetPEI and methylation modified siRNA (5 nmol) as indicated; primary Sertoli cells were isolated after 2 days, and ABP was determined by Western blot (H) and immunohistochemistry (J) using adjacent tissue. Densitometric analysis of ABP immunoblots is shown (I) (n = 3, *p < 0.05). Haematoxylin was used to stain the nuclei (blue). Black arrows: Sertoli cells.

Primary Sertoli cells were isolated from 18–22-day-old rats, and the cells isolated at this age had almost negligible somatic (i.e., Leydig cells) cell contamination^17^. We used oil red O and bodipy 493/503 to detect lipid droplets in Sertoli cells and used alkaline phosphatase staining to label germ cells. Lipid droplets were found in nearly all cells, but only a few cells were alkaline phosphatase positive (Fig. 1A), indicating a very high purity of the Sertoli cells used in this study.

To investigate the role of autophagy in the regulation of ABP expression, we treated primary rat Sertoli cells with chloroquine (CQ) or rapamycin, and found that CQ inhibited autophagy, while rapamycin enhanced autophagy, which is indicated by the LC3II/LC3I ratio (Fig. 1B). We then detected the ABP expression levels with both treatments, and we found that the suppression of autophagy promoted ABP expression, while stimulation of autophagy reduced the ABP expression (Fig. 1B, 1C, 1D). In addition, both treatments changed the ABP levels that Sertoli cells secreted to the supernatant (Fig. 1E). To further verify the results, we performed gene knockdown experiments with small interfering RNA (siRNA). The results demonstrated that inhibition of autophagy by ATG7 siRNA promoted ABP expression, while stimulation of autophagy by MTOR siRNA reduced ABP expression (Fig. 1F, 1G).

To extend our observations from cell culture, we performed intratesticular injection of siRNA for ATG7 or MTOR, and we found that *in vivo* results were consistent with those in primary cells (Fig. 1H, 1I, 1J). Inhibition or stimulation of autophagy by gene knockdown considerably changed the ABP expression levels in Sertoli cells in the seminiferous tubule (Fig. 1J).

### ABP colocalises with LC3 in primary rat Sertoli cells

**Figure 2:**
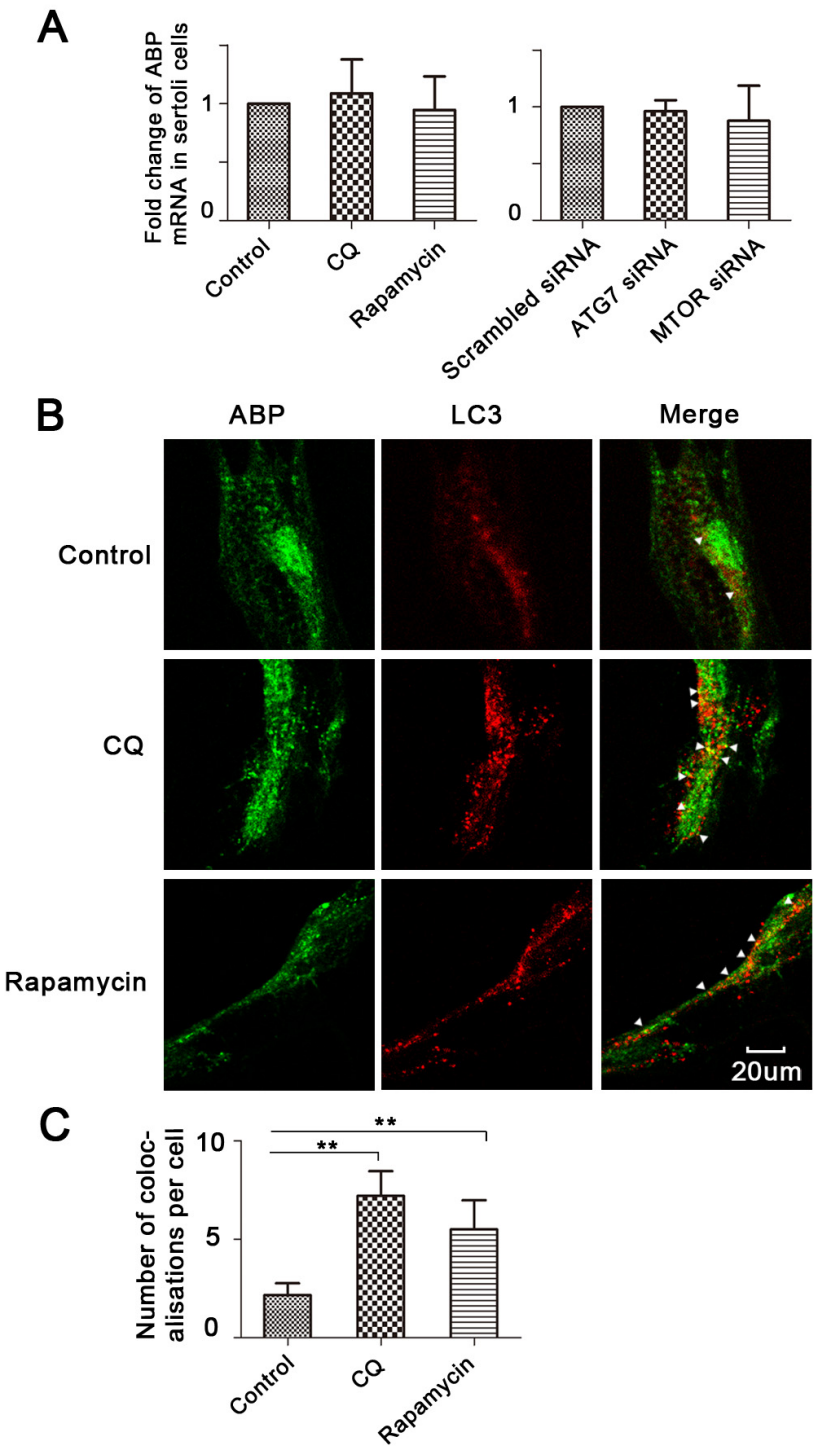
ABP colocalises with LC3 in primary Sertoli cells. (A), Rat primary Sertoli cells were treated with CQ (50 uM) or rapamycin (10 nM) for 24 h (n = 3), or treated with ATG7 or MTOR siRNA for 48 h (n = 3), ABP mRNA was determined by qPCR. (B–C), Cells were treated with CQ or rapamycin for 24 h, and ABP and LC3 were assessed with double immunofluorescence (B). The average number of colocalisations per cell was calculated from 10 random fields (approximately 200 cells) (C) (n = 3, **p < 0.01).

To investigate how autophagy regulates ABP expression, we first detected ABP gene transcription, and found that either inhibition or stimulation of autophagy did not change the ABP mRNA levels (Fig. 2A). We then used anti-ABP and anti-LC3 antibodies to identify their positions, and the results revealed that ABP colocalised with LC3 in primary rat Sertoli cells (Fig. 2B), indicating that ABP is engulfed by the autophagosomes and, therefore, degraded by lysosomes. We also found their colocalisation was promoted by either CQ or rapamycin treatment (Fig. 2B, 2C), suggesting that the ABP expression level is regulated by the autophagic flux.

### Clearance of ABP by autophagy is regulated by testosterone

**Figure 3:**
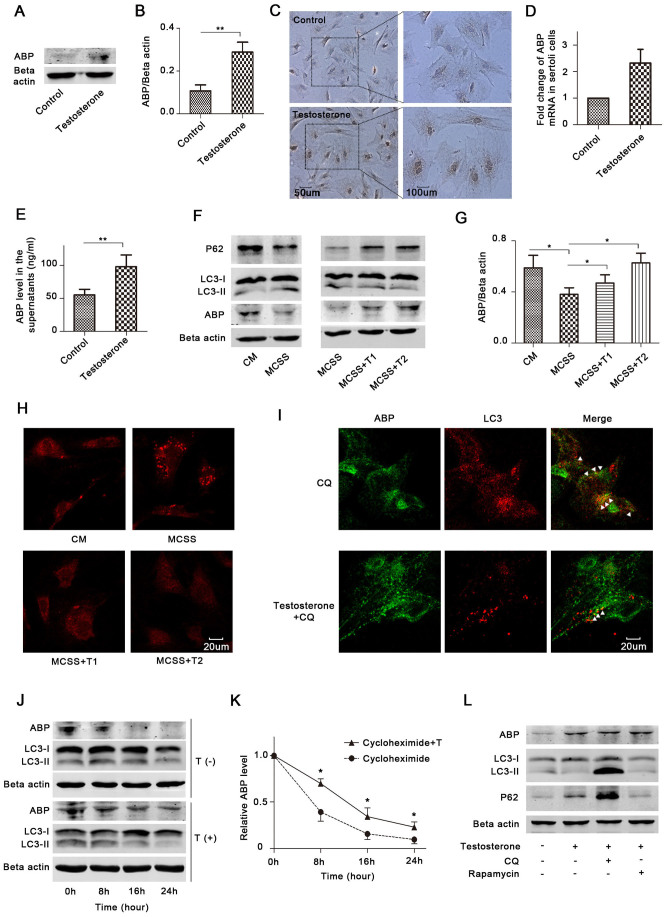
Testosterone up-regulates ABP expression through promoting its synthesis and suppressing autophagic degradation. (A–E), Rat primary Sertoli cells were cultured in CM and treated with testosterone for 24 h, and ABP expression in the cells was determined with Western blot (A), immunocytochemistry (C) and qPCR (D) (n = 3). The ABP expression level in the supernatant was assessed by ELISA (E) (n = 3, **p < 0.01). The densitometric analysis of ABP immunoblots is shown in (B) (n = 3, **p < 0.01). (F–H), Cells were cultured in CM, MCSS, MCSS+T1 and MCSS+T2 for 24 h, and LC3B, P62 and ABP were determined by Western blots (F). LC3B was also assessed by immunofluorescence (H). Densitometric analysis of the bands in ABP immunoblots is shown in (G) (n = 3, *p < 0.05). The blots of LC3B and Beta actin in Fig. 3F were cropped and full-length blots are presented in Supplementary Figure S4. The average number of LC3 puncta per cell was calculated in Supplementary Figure S5. (I), Cells were treated with CQ (50 uM) in the presence or absence of testosterone (10 nM), and the ABP and LC3 expression levels were assessed with double immunofluorescence after 24 h. The average number of ABP-LC3 colocalizations per cell was calculated in Supplementary Figure S7. (J–K), Cells were treated with cycloheximide (150 uM) in the presence or absence of testosterone (10 nM) as indicated (T: Testosterone) for different time periods, the ABP and LC3 expression were determined by Western blots (J). The relative ABP levels were determined by measuring the density of protein band, and normalized to Beta actin (K) (n = 3, *p < 0.05). The relative ABP protein level at time zero was termed as 1. The blots of ABP and Beta actin of the testosterone (+) group were cropped and full-length blots are presented in Supplementary Figure S10. (L), Cells were cultured in CM and treated with testosterone (10 nM), CQ (50 uM) or rapamycin (10 nM), as indicated. ABP, LC3B, P62 and Beta actin were determined by Western blots after 24 h. CM: Complete medium (medium with 10% foetal bovine serum); MCSS: Medium with 10% charcoal stripped serum. T1: Testosterone (10 nM); and T2: Testosterone (50 nM).

ABP production is promoted by testosterone^18^,^19^, and testosterone binds to ABP to stimulate its use. In addition, it has been reported that androgen might be involved in autophagy regulation^20^,^21^. We speculated that testosterone regulates ABP clearance via autophagy in Sertoli cells. Primary Sertoli cells were cultured in complete medium (CM) (medium containing 10% foetal bovine serum) and treated with or without testosterone; then, the ABP protein and mRNA expression were determined by Western blot (Fig. 3A, 3B), immunocytochemistry (Fig. 3C), and qPCR (Fig. 3D). ABP expression in the supernatants was also tested (Fig. 3E). The results showed that testosterone considerably increased the ABP mRNA and protein levels in primary cells, and testosterone promoted the secretion of ABP to the supernatant.

To detect whether testosterone regulates autophagy in Sertoli cells, we treated the cells with CM, medium with charcoal stripped serum (MCSS), and MCSS with testosterone. According to the Western blots of LC3B and P62 (P62 is as an adaptor protein in autophagy that interacts with LC3-II; it accumulates when autophagy is inhibited and decreases when autophagy is induced^22^), and immunofluorescence (LC3 puncta/cell), we found that removal of testosterone induced autophagy, while treatment with testosterone inhibited autophagy, and there were enhanced inhibitory effects when the concentration of testosterone was increased (Fig. 3F, 3H, Supplementary Fig. S1–S3, S5). We found the changes in P62 expression were more obvious than LC3B, to determine if P62 is testosterone responsive, we detected the P62 mRNA expression levels with different doses of testosterone. There were no substantial differences between the groups (Supplementary Fig. S6). We also detected the ABP levels by immunoblots in this experiment and found that testosterone-regulated autophagy affected the ABP expression levels (Fig. 3F, 3G). Besides, results from double immunofluorescence showed that testosterone reduced ABP colocalisation with LC3 under CQ treatment (Fig. 3I, Supplementary Fig. S7).

To provide direct evidence that testosterone regulates ABP degradation, we detected the ABP half-life in response to testosterone and/or cycloheximide treatment. Cycloheximide is a drug that can inhibit mRNA translation in the cells, and it is widely used in protein degradation analysis^23^,^24^. The results demonstrated cycloheximide alone didn't affect autophagy, while treatment with testosterone inhibited autophagy (Fig. 3J, Supplementary Fig. S8, S9). Besides, the half-life of ABP was about 6 h in primary Sertoli cells in the presence of cycloheximide alone, and it was increased to 10 h with testosterone treatment (Fig. 3J, 3K), suggesting that testosterone considerably decreased the ABP protein degradation.

To investigate how testosterone-regulated autophagy affects ABP expression, primary Sertoli cells were treated with testosterone in the presence of CQ or rapamycin. Interestingly, we found that either the inhibition or promotion of autophagy after testosterone treatment did not change ABP expression in Sertoli cells (Fig. 3L, Supplementary Fig. S11, S12), indicating that testosterone considerably and specifically suppressed the autophagic degradation of ABP.

### ABP clearance is independent of stress (hypoxia)-induced autophagy

**Figure 4:**
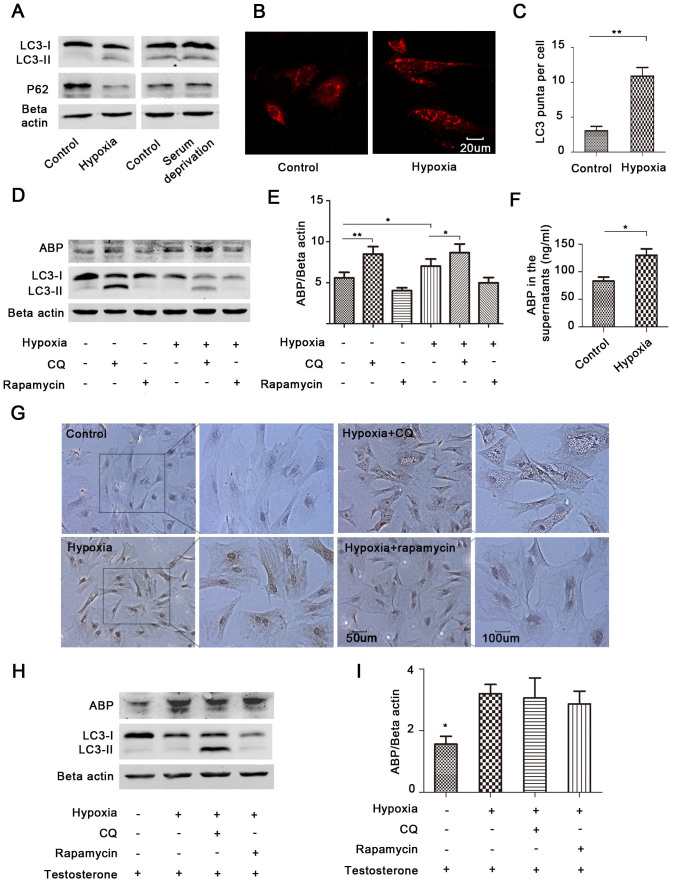
Stress-induced autophagy does not degrade ABP. (A–C), Primary Sertoli cells were cultured in hypoxia (1%) or serum deprivation medium for 24 h, LC3B and P62 were determined by Western blots (A). LC3B was also assessed by immunofluorescence (B). A high concentration of CQ (100 uM) was used before the immunofluorescence assay to promote the accumulation of LC3. The average number of puncta per cell was calculated from 10 random fields (C) (n = 3, **p < 0.01). (D–G), Cells were treated with CQ (50 uM) or rapamycin (10 nM) in the presence or absence of hypoxia for 24 h. ABP, LC3B and Beta actin were determined by Western blots (D). The bands in ABP immunoblots were analysed (E) (n = 3, *p < 0.05, **p < 0.01). ELISA was used to determine the ABP expression in the supernatants (F) (n = 3, *p < 0.05). ABP was also assessed by immunocytochemistry (G). (H–I), Cells were exposed to hypoxia and treated with testosterone (10 nM), CQ (50 uM) or rapamycin (10 nM) as indicated, ABP and LC3B were determined by immunoblots (H). The densitometric analysis of the bands in ABP immunoblots is shown in (I) (n = 3, *p < 0.05 when compared with any other group).

The above results allow us to speculate that the autophagic clearance of ABP is selectively regulated by testosterone. Hypoxia and/or nutrition deprivation induce autophagy in most cell types^25^,^26^. To investigate if stress induces autophagy in primary Sertoli cells, we treated cells with hypoxia or nutrition deprivation. The results showed that only hypoxia induced obvious autophagy in primary Sertoli cells (Fig. 4A), as indicated by the analysis of LC3B and P62 levels. Hypoxia can accelerate the turnover of LC3 protein; to promote the accumulation of LC3, a high concentration of CQ was used before the immunofluorescence assay in both the control and hypoxia groups, and we found that cells treated with hypoxia had more LC3 puncta (Fig. 4B, 4C).

It was recently reported that hypoxia up-regulates ABP (SHBG) in liver cells^27^. We also found that ABP expression in the primary Sertoli cells and their supernatant was considerably increased after exposure to hypoxia (Fig. 4D, 4E, 4F, 4G). To detect if hypoxia-induced autophagy participates in ABP regulation, we treated cells with hypoxia in the presence of CQ or rapamycin. The results showed that CQ further up-regulated ABP expression in hypoxia (Fig. 4D, 4E, 4G). However, the increase of ABP by CQ under hypoxia was less than that in normal conditions (Fig. 4E), indicating that the autophagic clearance of ABP under hypoxia was lower and that hypoxia-induced autophagy did not clear more ABP. To verify these results, we further treated the cells with testosterone, which inhibited the autophagic degradation of ABP (Fig. 3F). The results demonstrated that after testosterone treatment, neither inhibition nor promotion of hypoxia-induced autophagy changed ABP expression (Fig. 4H, 4I), suggesting that autophagy induced by hypoxia does not clear ABP and clearance of ABP is independent of hypoxia-induced autophagy.

## Discussion

In this study, both *in vitro* and *in vivo* experiments demonstrate that autophagy regulates ABP expression. Importantly, we found that autophagy reduces ABP expression through engulfing the protein, and the process seems to be selectively regulated by testosterone. Finally, stress (hypoxia)-induced autophagy does not change the ABP expression in primary Sertoli cells, which supports the idea that the autophagic degradation of ABP may be a selectively regulated process. To the best of our knowledge, we are the first to reveal that autophagy clears ABP in Sertoli cells; testosterone increases ABP expression not only by promoting its synthesis, and suppressing its autophagic degradation.

Sertoli cells are crucial support cells in the seminiferous tubule. To maintain a stable spermatogenic microenvironment and to support the growth of spermatogenic cells, Sertoli cells secrete many important proteins, such as anti-Müllerian hormone, inhibin, oestradiol and ABP. ABP can increase the concentration of testosterone in the seminiferous tubule to promote spermatogenesis. However, it has not previously been shown how ABP expression is regulated. Autophagy is an adaptive cellular response that is designed to protect the cell through eliminating cellular components by lysosomal degradation^28^. LC3 is the most widely monitored autophagy-related protein^29^. When autophagy is induced, there may be increases in the LC3-II/LC3-I ratios, decreases in both LC3-I and LC3-II, or a decrease in LC3-II relative to LC3-I if LC3-II degradation via lysosomal turnover is rapid. P62 is an adaptor protein that is required for the formation of ubiquitinated protein aggregates^30^. The interaction of P62 with both LC3 and ubiquitinated proteins can mediate the delivery of those aggregates to the autophagy system^31^. As a result, P62 accumulates when autophagy is inhibited, and it decreases when autophagy is induced^22^. The use of CQ to block autophagy in cultured cells is technically easy and has been widely accepted. CQ can inactivate lysosomal hydrolases by inhibiting lysosomal acidification, thereby increasing the LC3-II expression level and LC3-II/LC3-I ratios^32^. The MTOR inhibitor, rapamycin, can induce autophagy and promote the conversion of LC3-I to LC3-II, decreasing LC3-I expression and increasing the LC3-II/LC3-I ratios. Recent studies revealed that autophagy plays a central role in protein clearance and secretion^33^,^34^. In this context, we speculated that autophagy clears ABP in Sertoli cells, and, indeed we found that autophagy degrades ABP.

The conventional view about testosterone and ABP is that testosterone increases ABP synthesis and secretion^18^,^19^,^35^. However, until our study, it was unknown if testosterone regulates ABP clearance. Testosterone can bind to ABP to promote its use and reduce ABP accumulation in Sertoli cells. Also, it has been reported that androgen regulates autophagy through androgen receptor-mediated up-regulation of 78/BiP^20^; therefore, we speculated that testosterone may reduce the degradation of excess ABP through androgen receptor-mediated autophagy. Our results show that testosterone inhibits autophagy and up-regulates ABP expression. In addition, CQ or rapamycin can promote or inhibit ABP expression. However, after treatment with testosterone, ABP expression is not affected by CQ or rapamycin, indicating that testosterone might be a specific switch controller of the autophagic clearance of ABP. There might be a threshold, and when the testosterone concentration is below the threshold, the switch is turned on. Clearance of ABP by autophagy can be enhanced or inhibited by rapamycin or CQ. In contrast, adding testosterone up to the threshold turns off the switch, and the clearance of ABP is no longer affected by the drug that regulates autophagy. Therefore, it seems that the autophagic clearance of ABP is selectively regulated by testosterone.

Our results demonstrate the clearance of ABP is independent of stress (hypoxia)-induced autophagy, which further supports the idea that the autophagic degradation of ABP is a selectively controlled process. Autophagy can be stimulated by some stress factors, such as hypoxia or starvation^36^,^37^. Stress-induced autophagy degrades self proteins, maintains energy homeostasis and combats harmful factors^36^. However, in this study, we found that hypoxia-induced autophagy does not clear ABP. On the contrary, hypoxia up-regulates ABP expression. A possible explanation is that hypoxia increases testosterone through a VEGF pathway^38^,^39^, and testosterone stimulates ABP expression. Therefore, hypoxia might up-regulate ABP in an indirect way. It is possible that the induction of ABP by hypoxia is an adaptive response to hypoxia-induced cell death, maintaining a high concentration of ABP in the seminiferous tubule.

Autophagy is involved in spermatogenesis, and previous studies focused on the relationship between autophagy and germ cell death, sperm function, or testosterone secretion^40^,^41^,^42^. However, there is a paucity of data discussing autophagy and ABP in spermatogenesis. It has been reported that CQ has some toxic effects on reproductive function^43^, but the detailed mechanisms are still not clear. According to the results of this study, a possible reason is the dysregulation of ABP production induced by CQ.

In summary, we determined that in rat Sertoli cells, ABP can be cleared by autophagy. Of note, autophagy degrades ABP by engulfing the protein, and the process seems to be selectively regulated by testosterone, which can prolong ABP biological half-life by inhibiting autophagy. Finally, we found that ABP clearance is independent of stress (hypoxia)-induced autophagy. Although further studies are required to understand the role of autophagic clearance of ABP in Sertoli cells, these results have critical implications about the mechanisms by which testosterone regulates ABP expression as well as that by which ABP clearance is regulated.

## Methods

### Ethics statement

All procedures have been conducted according to the Declaration of Helsinki and NIH guidelines. All animal experiments were performed in accordance with the NIH Guide for the Care and Use of Laboratory Animals. The study was also approved by the institutional review board of Renji Hospital.

### Isolation and culturing of sertoli cells

Male Sprague–Dawley rats were purchased from Shanghai SLAC laboratory Animal Co., Ltd. A total of 20 Sprague–Dawley rats (18–22 days) were used for primary Sertoli cell isolation. The isolation method was as previously described with minor modifications^17^. Briefly, after the tunica albuginea was removed, all testes were placed into F12/DMEM (1:1) (11039, GIBCO) and washed 2 times. The testes were cut into ~1-mm pieces and washed to remove contaminating blood cells, they were then centrifuged at 800 *g* for 2 min. Testes were resuspended in F12/DMEM with 0.1% trypsin (25300, GIBCO) and then placed in a shaking water bath at 60 osc/min for 30 min, to release Leydig cells. The bottom seminiferous tubules were transferred to a vial with F12/DMEM supplemented with 10% foetal bovine serum (10099, Gibco) and washed until F12/DMEM was clear. The supernatant was discarded and, cells were resuspended in F12/DMEM containing 0.1% collagenase V (C9263, Sigma-Aldrich) and placed in a shaking water bath at 60 osc/min for 40 min, to remove peritubular myoid cells. Cells were washed by centrifugation at 800 g two times, resuspended cells with F12/DMEM containing 0.1% hyaluronidase (H3506, Sigma-Aldrich) and placed in the shaking water bath for 30 min, to break down hyaluronic acid. Cells were washed twice by centrifugation at 800 *g*, resuspended and incubated with F12/DMEM supplemented with 50 U/ml penicillin, 50 μg/ml streptomycin (10378, GIBCO), and 10% charcoal stripped foetal bovine serum (12676, GIBCO) or 10% foetal bovine serum. Forty-eight hours after plating, cells were treated with 20 mM Tris (pH 7.4) for 2.5 min to remove residual germ cells. After two washes, cells were incubated in a humidified CO2 incubator at 35°C with 95% air and 5% CO2. If cells were cultured in hypoxia, the oxygen was maintained at 1%.

### Chemical Reagents, siRNA, *in vitro* and *in vivo* Transfection

Chloroquine (CQ), oil red O, testosterone and rapamycin were obtained from Sigma-Aldrich, MO, USA (C6628, O0625, T6147, 37094). Cycloheximide was purchased from Chengdu Ai Keda Chemical Technology Company (66-81-9). Methylation modified siRNA against rat ATG7 (5′-GGAUACAAGCUUGGCUGCUACUUCU-3′) and MTOR (5′-CGGCAGACUGGCUCUUGCUCAUAAA-3′) were purchased from RiboBio, Guangzhou, China. Transfection of nucleic acids was performed using Lipofectamine 2000 (11668, Life Technologies) and in vivo-jetPEI (201-10G, Polyplus) according to the manufacturer's instructions. For intra-testicular injection, the rats (8 weeks) were randomly divided into 3 groups (Scrambled siRNA group, n = 3; ATG7 siRNA group, n = 3; and MTOR siRNA group, n = 3). A mixed liquid (50 ul) with 5 nmol siRNA and in vivo-jetPEI was directly injected into the rat testis according to the manufacturer's instructions. Rats were sacrificed after 2 days, and ABP was determined by immunohistochemistry in testis tissue. Primary Sertoli cells from the adjacent testis tissue were also isolated, and ABP, ATG7, MTOR, and LC3B were detected with Western blots analysis.

### RNA Isolation, cDNA Synthesis and Quantitative Real-time PCR (qPCR)

Total RNA isolation, cDNA synthesis and qPCR were performed as previously described^44^,^45^. The primers for qPCR are listed as follows: rat GAPDH (5′-3′) F: GGCACAGTCAAGGCTGAGAATG, R: ATGGTGGTGAAGACGCCAGTA; rat SHBG (ABP) (5′-3′) F: ATGTGGACCTGCAACCTGGAC, R: TGCTCCATCCACCAGCTTAAATC and; rat SQSTM1 (P62) (5′-3′) F: CCTCAGCCCTCTAGGCATCG, R: GTACAGGGCAGCTTCCTTCA.

### Protein extraction and western blot

Protein extraction and Western blot were performed as previously described^26^. Protein from primary Sertoli cells was isolated with M-PER Mammalian Protein Extraction Reagent (78501, Thermo Scientific). Rabbit anti-LC3B (L7543), ACTB (beta actin) (8457), P62/SQSTM1 (5114), ATG7 (8558) and MTOR (2983) were purchased from Sigma-Aldrich Co. LLC. Anti-SHBG (ABP) (SC-32891) was obtained from Santa Cruz Biotechnology, Inc.

### Oil red O, Bodipy 493/503 and Alkaline phosphatase staining

For oil red O and bodipy 493/503 staining, cells were washed with PBS, fixed with 4% formaldehyde for 20 min, and stained with oil red O (0.2%) or bodipy 493/503 (D3922, Life Technologies. stock concentration 2 mg/ml, working solution 1:200 dilution) for 15 min at room temperature. The alkaline phosphatase kit was purchased from Shanghai Fushen Biotechnology Company and, staining was performed according to the manufacturer's instructions.

### Immunofluorescence and confocal microscopy

Cells were washed with PBS and fixed in ice-cold ethanol for 15 min at −20°C. After blocking for 20 min, cells were incubated in the primary antibodies at 4°C overnight, and Alexa Fluor 594 goat anti-mouse IgG (A11012, Invitrogen) and Alexa Fluor 488 goat anti-rabbit IgG (A11008, Invitrogen) served as the secondary antibodies. To prepare the glass histological slide, rat testis was fixed in Bouin's fixative, embedded in paraffin, and sectioned at 3 μm thickness. Anti-SHBG (ABP) (sc-32891) and Anti-LC3B (sc-376404) were purchased from Santa Cruz Biotechnology, Inc. Immunofluorescence was detected with confocal microscopy as previously described^26^.

### Immunocytochemistry, Immunohistochemistry and Enzyme-Linked Immunosorbent Assays (ELISA)

Immunocytochemistry and immunohistochemistry was performed as previously described^46^. Briefly, cells or tissue were incubated in the primary antibodies at 4°C overnight and the secondary antibodies for 30 min at 37°C; then, they were visualized with diaminobenzadine (DAB) and counterstained with hematoxylin. The ABP levels in the supernatants were assessed by an ELISA kit for rats (CSB-E12118r, Cusabio) according to the manufacturer's instructions.

### Statistical analysis

Data analyses were conducted with SPSS 16.0 (SPSS, IL, USA). The bar charts were plotted with GraphPad-Prism5 (GraphPad, CA, USA). Data are presented as the mean ± standard deviation (SD) and analysed by Student's t test. All tests were 2-tailed, and p < 0.05 was considered statistically significant. Each experiment was performed in triplicate.

## Author Contributions

Y.M., L.M.X. and H.Z.Y. performed experiments. Y.M., X.N.K. and H.L.D. conceived of the study. Y.M., Y.R.H. and X.N.K. participated in the design of this study. X.N.K., Y.R.H. and H.L.D. supervised the work. Y.M. and X.N.K. analyzed data. The manuscript was drafted by Y.M., and reviewed by all authors. All authors approved the final version of the manuscript to be published.

## Supplementary Material

Supplementary Information Supplementary Figures
