# Supplementary material for: Testosterone regulates the autophagic clearance of androgen binding protein in rat Sertoli cells
Source: Sci Rep. 2015 Mar 9;5:8894. doi: 10.1038/srep08894 (PMC4352847; doi:10.1038/srep08894)
Supplement: Supplementary Information — Supplementary Figures [file srep08894-s1.doc]

**Testosterone regulates the autophagic clearance of androgen binding protein in rat sertoli cells**

Yi Ma, Hao-Zheng Yang, Long-Mei Xu, Yi-Ran Huang, Hui-Li Dai, Xiao-Nan Kang


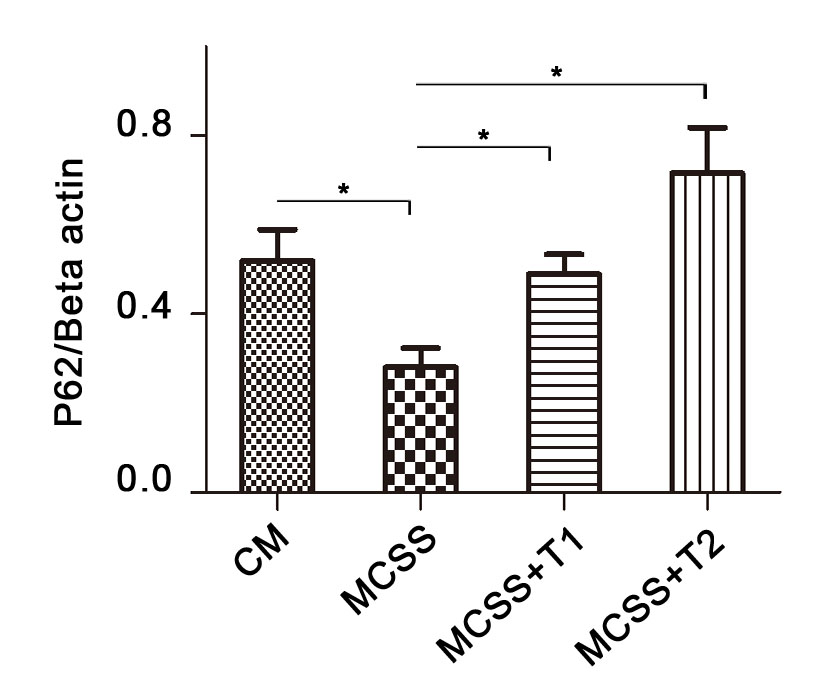


Figures S1. Densitometric analysis of p62 immunoblots in Figure 3F. n=3, *p<0.05 CM: Complete medium (medium with 10% foetal bovine serum); MCSS: Medium with 10% charcoal stripped serum. T1: Testosterone (10 nM); and T2: Testosterone (50 nM).


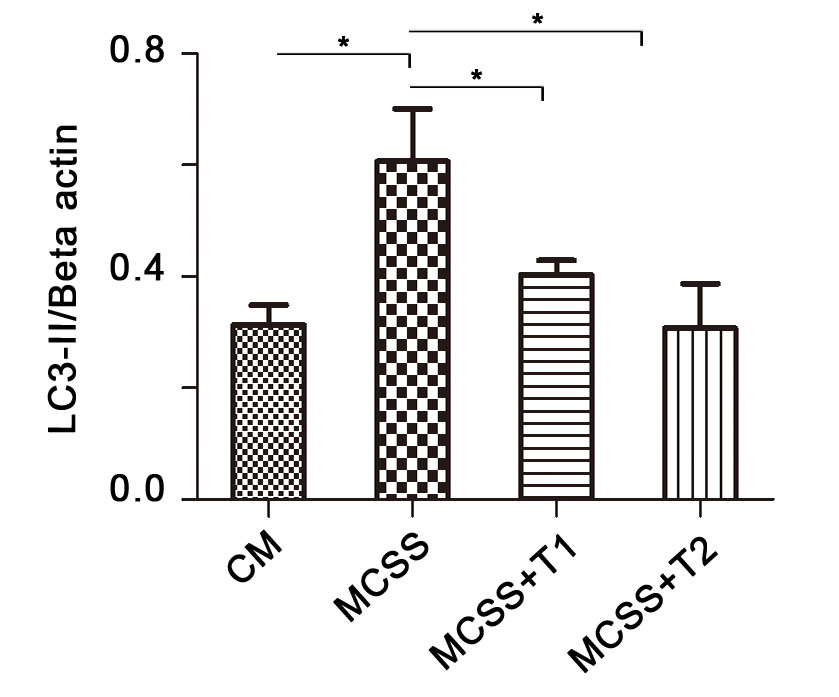


Figures S2. Densitometric analysis of LC3-II immunoblots in Figure 3F. n=3, *p<0.05 CM: Complete medium (medium with 10% foetal bovine serum); MCSS: Medium with 10% charcoal stripped serum. T1: Testosterone (10 nM); and T2: Testosterone (50 nM).


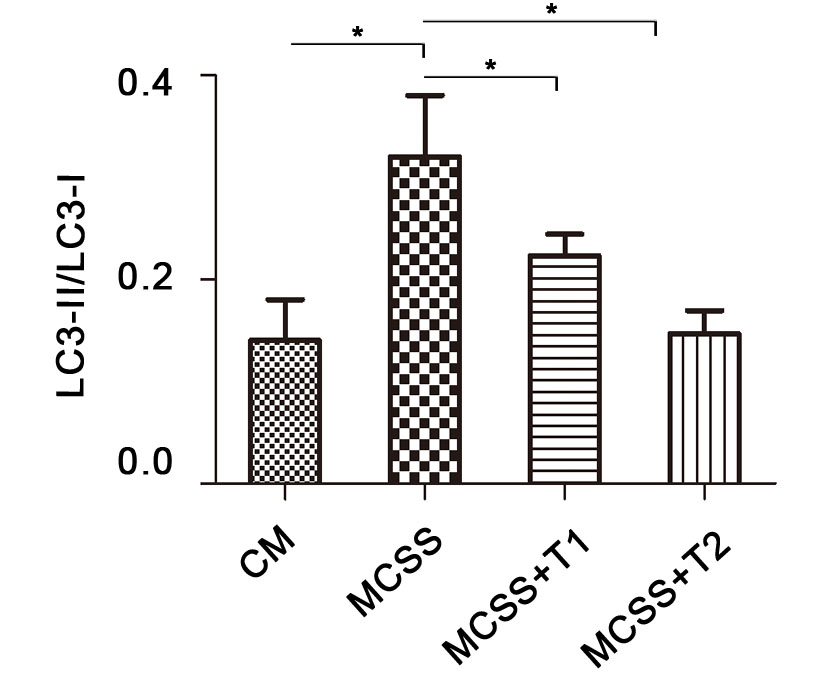


Figures S3. Densitometric analysis of LC3-II and LC3-I immunoblots in Figure 3F. n=3, *p<0.05 CM: Complete medium (medium with 10% foetal bovine serum); MCSS: Medium with 10% charcoal stripped serum. T1: Testosterone (10 nM); and T2: Testosterone (50 nM).


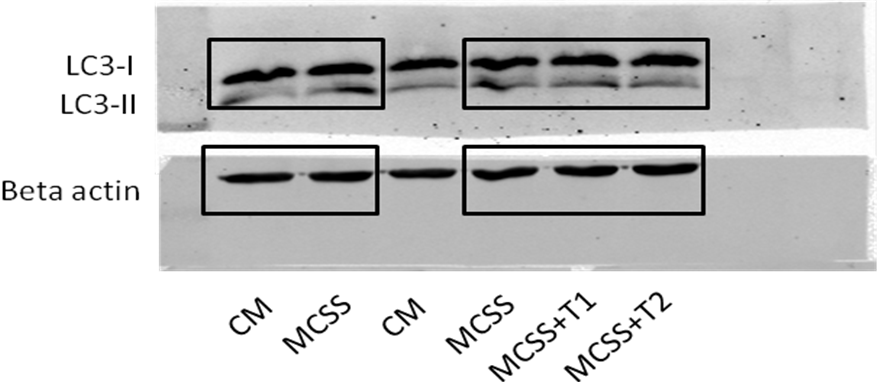


Figures S4. Full-length blots of Figure 3F (LC3B and Beta actin). CM: Complete medium (medium with 10% foetal bovine serum); MCSS: Medium with 10% charcoal stripped serum. T1: Testosterone (10 nM); and T2: Testosterone (50 nM).


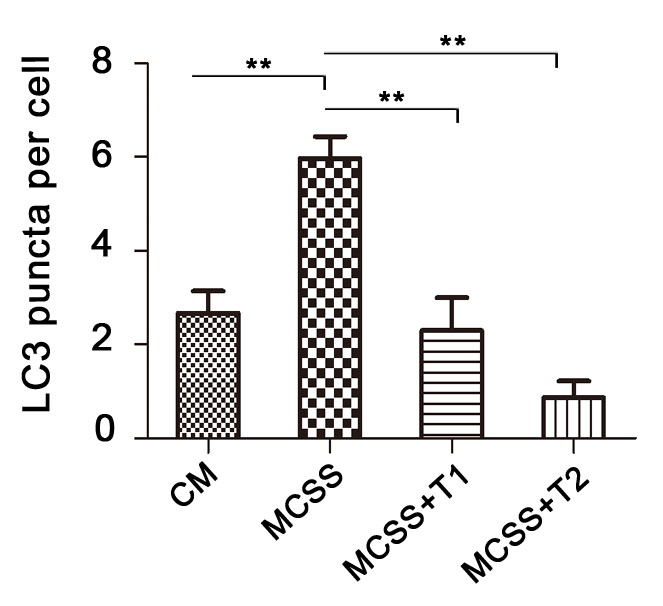


Figures S5. The average number of LC3 puncta per cell in Figure 3H. n=3, **p<0.01 CM: Complete medium (medium with 10% foetal bovine serum); MCSS: Medium with 10% charcoal stripped serum. T1: Testosterone (10 nM); and T2: Testosterone (50 nM).


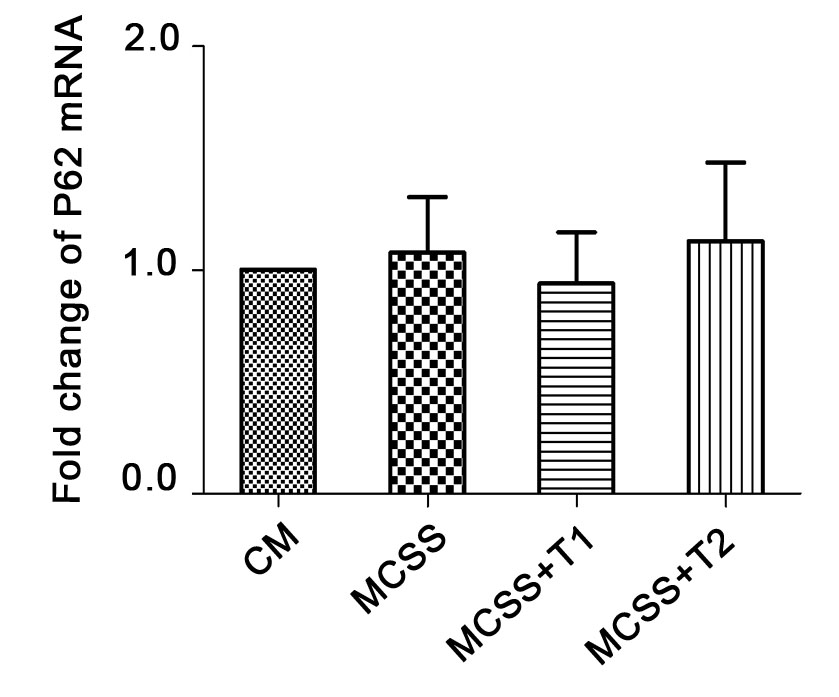


Figures S6. P62 mRNA changes in response to testosterone treatment. CM: Complete medium (medium with 10% foetal bovine serum); MCSS: Medium with 10% charcoal stripped serum. T1: Testosterone (10 nM); and T2: Testosterone (50 nM).


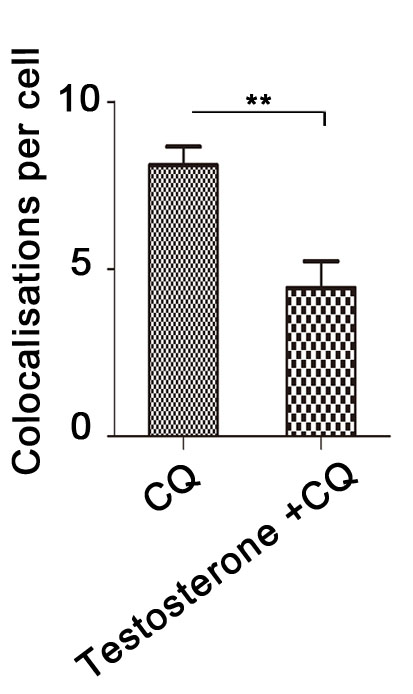


Figures S7. The average number of ABP-LC3 colocalisations per cell in Figure 3I. n=3, **p<0.01


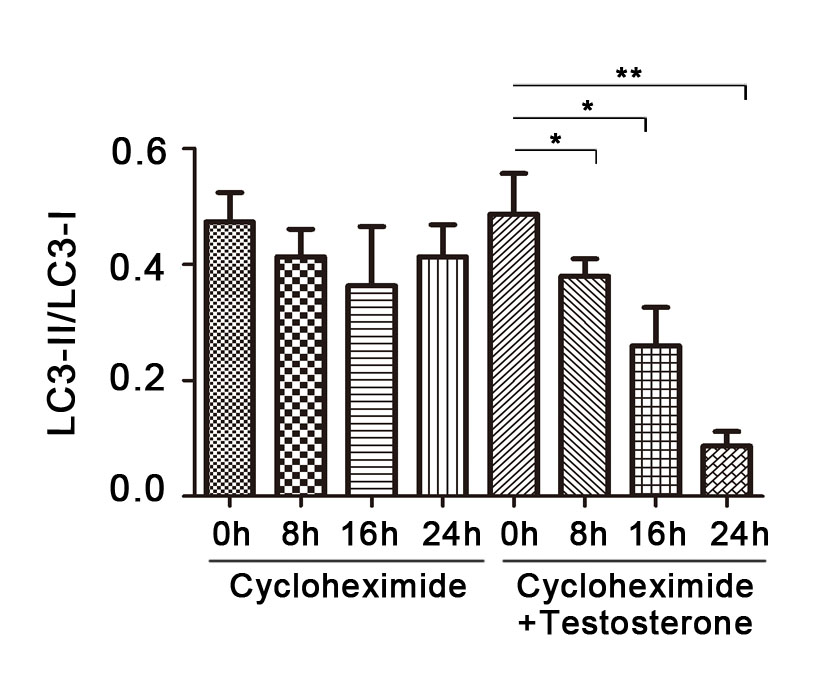


Figures S8. Densitometric analysis of LC3-II and LC3-I immunoblots in Figure 3J. n=3, *p<0.05, **p<0.01


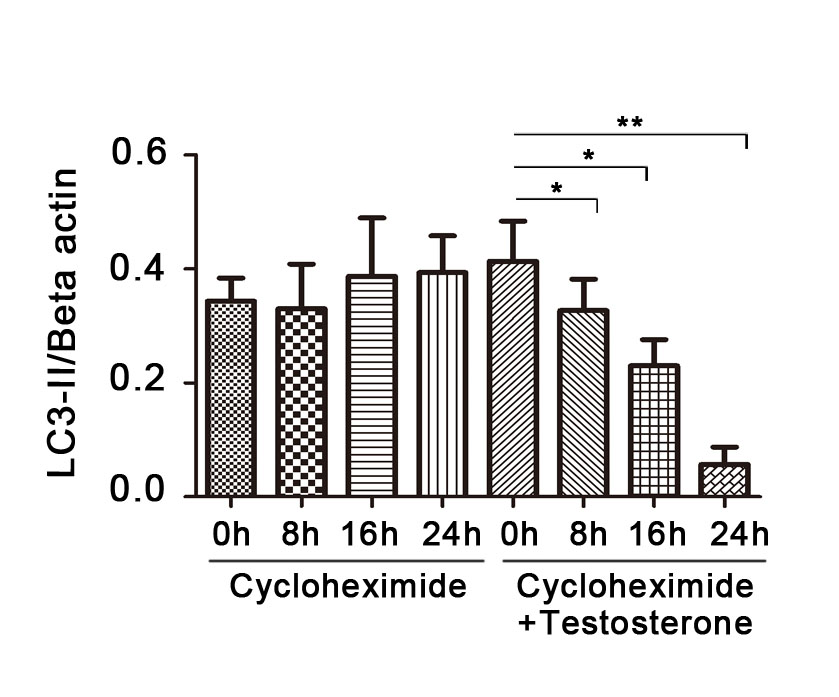


Figures S9. Densitometric analysis of LC3-II immunoblots in Figure 3J. n=3, *p<0.05, **p<0.01


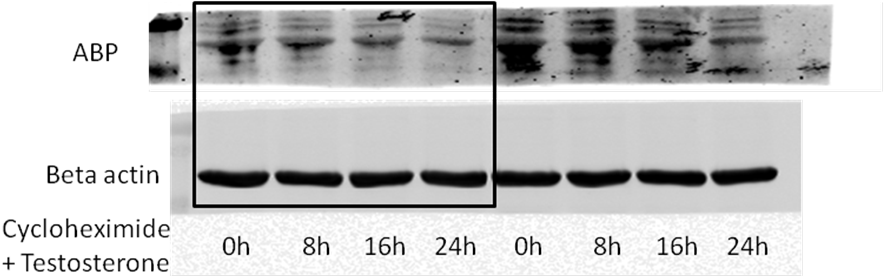


Figures S10. Full-length blots of ABP and Beta actin of the testosterone (+) group in Figure 3J.


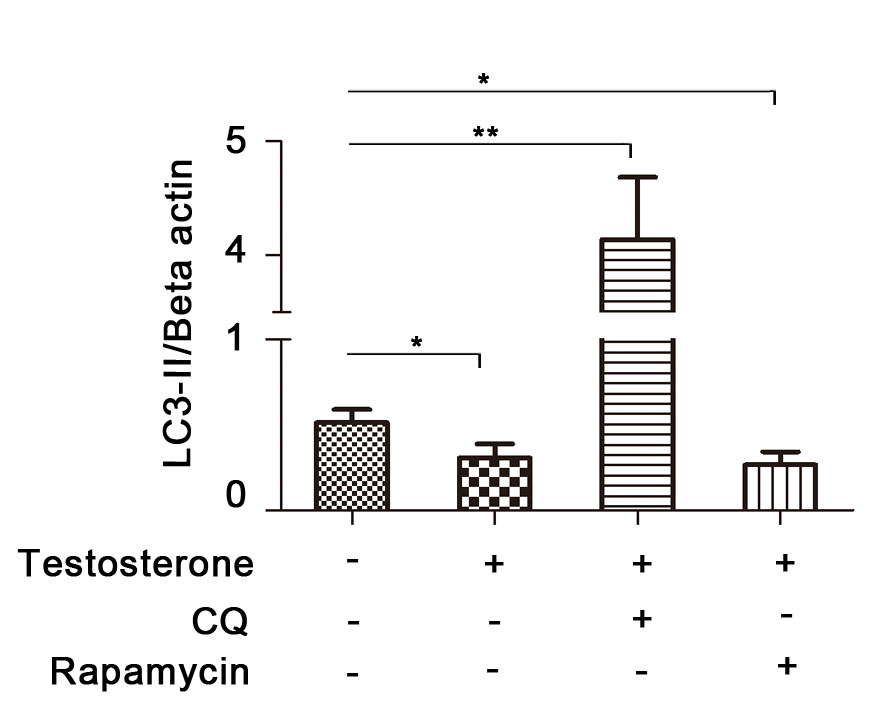


Figures S11. Densitometric analysis of LC3-II immunoblots in Figure 3L. n=3, *p<0.05, **p<0.01


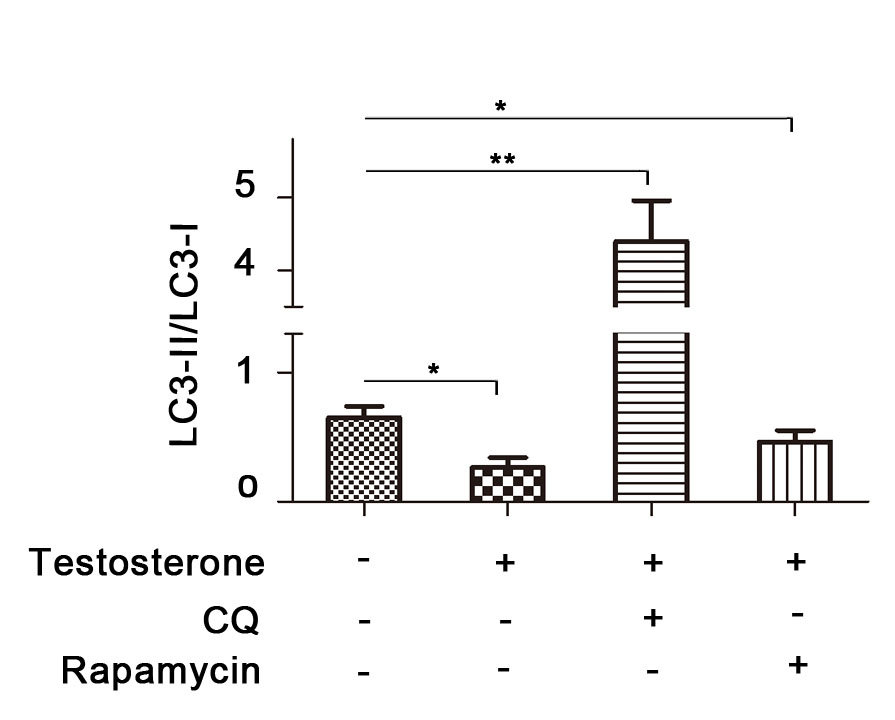


Figures S12. Densitometric analysis of LC3-II and LC3-I immunoblots in Figure 3L. n=3, *p<0.05, **p<0.01
